# Anti-Cancer Effects of Protein Extracts from *Calvatia lilacina*, *Pleurotus ostreatus* and *Volvariella volvacea*

**DOI:** 10.1093/ecam/neq057

**Published:** 2011-06-18

**Authors:** Jin-Yi Wu, Chi-Hung Chen, Wen-Huei Chang, King-Thom Chung, Yi-Wen Liu, Fung-Jou Lu, Ching-Hsein Chen

**Affiliations:** ^1^Graduate Institute of Biomedical and Biopharmaceutical Sciences, College of Life Sciences, National Chiayi University, A25-303 Room, Life Sciences Hall, 300 Syuefu Road, Chiayi 60004, Taiwan; ^2^Graduate Institute of Food Science and Biopharmaceutics, National Chiayi University, Chiayi, Taiwan; ^3^Department of Chemical Biology, National Pingtung University of Education, Pingtung, Taiwan; ^4^Department of Biology, The University of Memphis, Memphis, TN, USA; ^5^Department of Applied Chemistry, Chung Shan Medical University, Taichung, Taiwan

## Abstract

*Calvatia lilacina* (CL), *Pleurotus ostreatus* (PO) and *Volvariella volvacea* (VV) are widely distributed worldwide and commonly eaten as mushrooms. In this study, cell viabilities were evaluated for a human colorectal adenocarcinoma cell line (SW480 cells) and a human monocytic leukemia cell line (THP-1 cells). Apoptotic mechanisms induced by the protein extracts of PO and VV were evaluated for SW480 cells. The viabilities of THP-1 and SW480 cells decreased in a concentration-dependent manner after 24 h of treatment with the protein extracts of CL, PO or VV. Apoptosis analysis revealed that the percentage of SW480 cells in the SubG_1_ phase (a marker of apoptosis) was increased upon PO and VV protein-extract treatments, indicating that oligonucleosomal DNA fragmentation existed concomitantly with cellular death. The PO and VV protein extracts induced reactive oxygen species (ROS) production, glutathione (GSH) depletion and mitochondrial transmembrane potential (ΔΨ_m_) loss in SW480 cells. Pretreatment with *N*-acetylcysteine, GSH or cyclosporine A partially prevented the apoptosis induced by PO protein extracts, but not that induced by VV extracts, in SW480 cells. The protein extracts of CL, PO and VV exhibited therapeutic efficacy against human colorectal adenocarcinoma cells and human monocytic leukemia cells. The PO protein extracts induced apoptosis in SW480 cells partially through ROS production, GSH depletion and mitochondrial dysfunction. Therefore, the protein extracts of these mushrooms could be considered an important source of new anti-cancer drugs.

## 1. Introduction

*Calvatia lilacina* (CL), *Pleurotus ostreatus* (PO) and *Volvariella volvacea* (VV) are three common medicinal or edible mushrooms. Among them, CL was widely used by CAM practitioners in China and Taiwan for treatment of traumatic bleeding, hemorrhagic vomiting, nasal bleeding, throat and nasopharyngeal pain, loss of voice and coughing. PO showed antimicrobial and antineoplastic activities [1]. Our previous study demonstrated that the protein extracts of CL induce apoptosis through glutathione (GSH) depletion in human colorectal cancer SW480 cells [2]. The extraction of PO suppresses the proliferation of breast and colon cancer cells via p53-dependent and p53-independent pathways [3]. The water-soluble polysaccharide of PO is capable of enhancing concanavalin A- or lipopolysaccharide-induced lymphocyte proliferation [4, 5] and induces apoptosis in human PC-3 androgen-independent cancer cells as well as human HT-29 colon cancer cells [6, 7]. DNA isolated from the fruit body of PO augmented NK cytotoxic activity *in vitro* and significantly increased the life span of mice with solid Ehrlich carcinoma [8]. Despite the observation of anti-cancer effects for PO, the effects of the protein extract of PO on cancer are unclear thus far; moreover, there have been no anti-cancer studies on VV protein extracts. The anti-cancer effects of the protein extracts of PO and VV are worth studying. Colorectal cancer and leukemia are two common cancers in human. The aim of this study was to evaluate the anti-cancer effects of three protein extracts from CL, PO and VV on human colorectal cancer SW480 cells and monocytic leukemia THP-1 cells. The relationship between apoptosis and intracellular oxidative stress in cancer cells treated with PO and VV protein extracts was also evaluated.

## 2. Methods

### 2.1. Experimental Material

CL was collected from the National Chiayi University, Chiayi, Taiwan, in May 2008. PO and VV were bought from Carrefour Supermarket, Chiayi, Taiwan, in June 2008.

### 2.2. Extraction and Isolation of Proteins

CL, PO and VV (400–450 g) were washed twice with distilled water and then homogenized in cold extraction buffer (50 mM KH_2_PO_4_, 150 mM NaCl and 1 mM EDTA, pH 7.3) at maximum speed in a Waring blender for 10 min. The water-soluble protein extract homogenate was filtered through two layers of surgical gauze, and the filtrate was centrifuged at 8900 g for 20 min. The supernatants were precipitated by addition of ammonium sulphate to 80% saturation, incubated at 4°C overnight, and then centrifuged at 8900 g for 30 min. The protein pellets were resuspended in a phosphate buffer solution (123 mM NaCl, 10.4 mM Na_2_HPO_4_ and 3.16 mM KH_2_PO_4_, pH 7.3) (weight/volume = 1 : 1) and centrifuged. The supernatants were dialyzed against a 50-fold volume of phosphate buffer solution three times at 4°C and clarified by centrifugation. The supernatants were filtered through 0.45 *μ*m Millex filter units (Millipore) and concentrated using Amicon Ultra-4 (10 kDa cut-off, Millipore) tubes at 4000 g for 20 min. The 1 mL concentrate was sterilized using 0.22 *μ*m Millex filter units (Millipore) in a laminar flow chamber; it was then freeze-dried to obtain a powder and stored at −80°C as stock. The Bio-Rad protein assay reagent (Bio-Rad Lab., Hercules, CA), 12% sodium dodecyl sulphate polyacrylamide gel electrophoresis (SDS-PAGE) and Coomassie Brilliant Blue staining were utilized for the detection of CL, PO and VV proteins.

### 2.3. Cell Lines and Reagents

The human colorectal cancer cell line SW480 and the monocytic leukemia cell line THP-1 were obtained from the Bioresource Collection and Research Center (Hsinchu, Taiwan, ROC). Dulbecco's modified Eagle's medium (DMEM), RPMI-1640 and foetal bovine serum (FBS) were obtained from Hyclone (South Logan, UT, USA). Chloromethylflourescein diacetate (CMF-DA) was acquired from Invitrogen Corporation (Carlsbad, CA, USA). Propidium iodide (PI), *N*-acetylcysteine (NAC), GSH, cyclosporine A (CyA), 2′,7′-dichlorodihydrofluorescein-diacetate (DCFH-DA), rhodamine 123, 3-(4,5-dimethylthiazol-2-yl)-2,5-diphenyl tetrazolium bromide (MTT), DNase-free RNase A and other chemicals were purchased from Sigma Chemical Co. (St. Louis, MO, USA).

### 2.4. Cell Culture and Treatment

SW480 and THP-1 cells were cultured in DMEM and RPMI-1640 medium, respectively, containing 10% FBS. The stock solutions of CL, PO and VV were dissolved in the phosphate buffer saline (PBS) (136.89 mM NaCl, 2.68 mM KCl, 10.14 mM Na_2_HPO_4_ and 1.76 mM KH_2_PO_4_, pH 7.4), and different concentrations (*μ*g mL^−1^) were prepared in DMEM or RPMI-1640 media.

### 2.5. Cell Viability Assays

Cell viability was evaluated using the MTT assay. Yellow MTT was reduced to purple formazan by dehydrogenases in the mitochondria of living cells. A solubilization agent, such as dimethyl sulfoxide (DMSO) or SDS, was used to dissolve the insoluble purple formazan product into a colored solution. The absorbance of this colored solution was quantified at a certain wavelength (usually 570 nm) with a spectrophotometer. The effectiveness of the protein extracts in causing cell death was evaluated by comparing the amount of purple formazan produced by cells treated with the extracts to that produced by untreated cells. Cells (1 × 10^6^) were cultured in 60 mm tissue-culture dishes for 24 h. The culture medium was replaced with a new medium and the cells were exposed to various concentrations of CL, PO or VV for 24 h. After the indicated treatments, cells were incubated for 2 h with 0.5 mg mL^−1^ of MTT reagent and then lysed with DMSO. Absorbance was measured at 570 nm in a microplate reader (Bio-Rad, Richmond, CA, USA).

### 2.6. Cell Cycle and DNA Damage Analysis

The cell cycle and DNA damage were evaluated with PI staining and flow cytometry. PI is a specific fluorescent dye that stains the double-stranded DNA. In methanol-fixed cells, the PI molecules translocate into the nucleus and bind to the double-stranded DNA. Using flow cytometry, PI was excited at 488 nm and emitted fluorescence at 580 ∼ 630 nm. The G_1_ and G_2_/M phases are represented by the diploid and the tetraploid regions, respectively, of the DNA distribution histograms. Cells in the S phase were found between the diploid and the tetraploid regions. The length of double-stranded DNA in cells with DNA damage was smaller than that of cells in the G_1_ phase. Fewer PI molecules were found in DNA-damaged cells as compared with the G_1_ phase; the fluorescent intensity of PI in DNA-damaged cells was weaker than that of cells in the G_1_ phase. The percentage of DNA-damaged cells was characterized as the percentage of cells in the SubG_1_ region of the DNA distribution histograms. Cells (1 × 10^6^) were cultured in 60-mm tissue-culture dishes for 24 h. The culture medium was replaced by a new medium, and then cells were exposed or not to various concentrations of the protein extracts of PO and VV for 24 h. After treatment, adherent and floating cells were pooled, washed with PBS, fixed in PBS-methanol (1 : 2, volume/volume) solution, and then maintained at 4°C for at least 18 h. After one wash with PBS, the cell pellets were stained with a fluorescent probe solution containing PBS, 50 *μ*g mL^−1^ PI and 50 *μ*g mL^−1^ DNase-free RNase A for 30 min at room temperature in the dark. The DNA fluorescence of PI-stained cells was analyzed by excitation at 488 nm and monitored through a 630/22 nm band-pass filter using a Becton-Dickinson FACScan flow cytometer (Franklin Lakes, NJ, USA). A minimum of 10 000 cells was counted per sample, and the DNA histograms were evaluated further using Modfit software on a PC workstation to calculate the percentage of cells in various phases of the cell cycle.

### 2.7. Measurement of Intracellular Reactive Oxygen Species by Flow Cytometry

Production of intracellular Reactive oxygen species (ROS) was detected by flow cytometry using DCFH-DA. DCFH-DA is a ROS probe that undergoes intracellular deacetylation to DCFH by esterase in cytosol, followed by ROS-mediated oxidation to a DCF fluorescent species that is excited at 485 nm and emits fluorescence at 530 nm. DCFH-DA can be used to measure ROS generation in the cytoplasm and in cellular organelles such as the mitochondria. Fluorescence intensity is quantified using flow cytometry or a microplate spectrophotometer. After treatment, cells were treated with 20 *μ*M DCFH-DA for 30 min in the dark, washed once with PBS, detached by trypsinization, collected by centrifugation and suspended in PBS. The intracellular ROS levels, as indicated by the fluorescence of dichlorofluorescein (DCF), were measured with a Becton-Dickinson FACScan flow cytometer.

### 2.8. Measurement of GSH Depletion

The intracellular GSH levels were evaluated using CMF-DA. After treatment, the cells were incubated with 25 *μ*M CMF-DA for 20 min at 37°C in a CO_2_ incubator. Once CMF-DA is internalized into the cell, cytosolic esterase cleaves the acetates, and the chloromethyl group reacts with intracellular thiols, altering the probe into a cell-impermeable fluorescent dye-thioether adduct, CMF. The CMF fluorescence is directly associated with intracellular GSH level. After CMF-DA staining, the cells were washed with PBS, collected by centrifugation and then fluorescence was measured with a Becton-Dickinson FACSan flow cytometer.

### 2.9. Measurement of ΔΨ_m_ by Flow Cytometry

After treatment, the cells were trypsinized and then incubated with 5 *μ*M rhodamine 123 for 30 min at 37°C in a CO_2_ incubator. Rhodamine 123, a lipophilic cation, is specifically and selectively internalized into the mitochondria of mammalian cells. The collection of rhodamine 123 is dependent on the proton gradient produced across mitochondrial membranes. The accumulation of rhodamine 123 within cells can thus be used as an indicator of mitochondrial membrane potential and mitochondrial function. Following the incubation step, ΔΨ_m_, as indicated by the change in the fluorescence level of rhodamine 123, was analyzed using a Becton-Dickinson FACScan flow cytometer.

### 2.10. Statistical Analysis

Data are presented as means ± SD from at least three independent experiments and were analyzed using Student's *t-*test. *P* < .05 was considered as statistically significant [9].

## 3. Results

### 3.1. The Number of Major Proteins and Their Molecular Weights Were Evaluated in the Protein Extracts of CL, PO and VV

**Figure 1:**
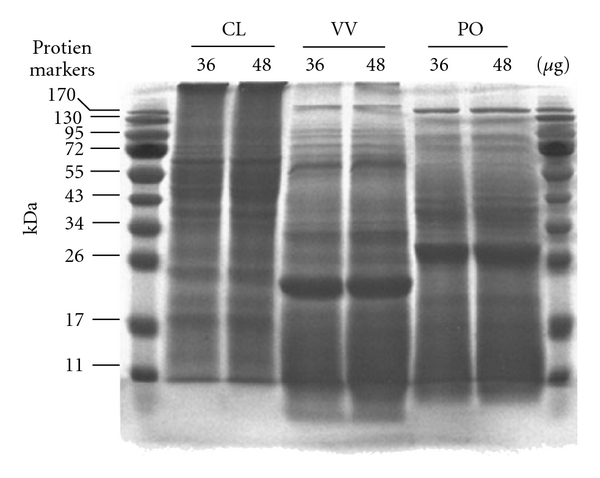
Protein extract analysis for CL, VV and PO. Concentrations of proteins in extracts of CL, VV and PO were quantified with Bio-Rad protein assay reagent. BSA was used as a standard for protein analysis. Protein extracts (36 and 48 *μ*g) of CL, VV and PO were subjected to SDS-PAGE (12% (w/v) polyacrylamide). CL, VV and PO protein extracts were stained with Coomassie Brilliant Blue. The positions of molecular mass markers (in kilodaltons) are shown on the left and on the right.

To detect the number of major proteins and their molecular weights in the protein extracts of CL, PO and VV, 12% SDS-PAGE gel electrophoresis and Coomassie Brilliant Blue staining were used. The profiles of protein extracts from CL, PO and VV used in this study are presented in Figure 1. Major proteins with various molecular weights around 70, 55, 45, 40, 28, 24, 20, 18 and 11 kDa were visualized after Coomassie Brilliant Blue staining in 36 and 48 *μ*g of protein extracts of CL. Major proteins with various molecular weights around 175, 96, 95, 72, 68, 32, 23 and 11 kDa were visualized in 36 and 48 *μ*g of protein extracts of VV. Major proteins with various molecular weights around 170, 130, 95, 72, 43, 42, 28, 20 and 11 kDa were visualized in 36 and 48 *μ*g of protein extracts of PO.

### 3.2. CL, PO and VV Decreased the Viabilities of THP-1 and SW480 Cells

**Figure 2:**
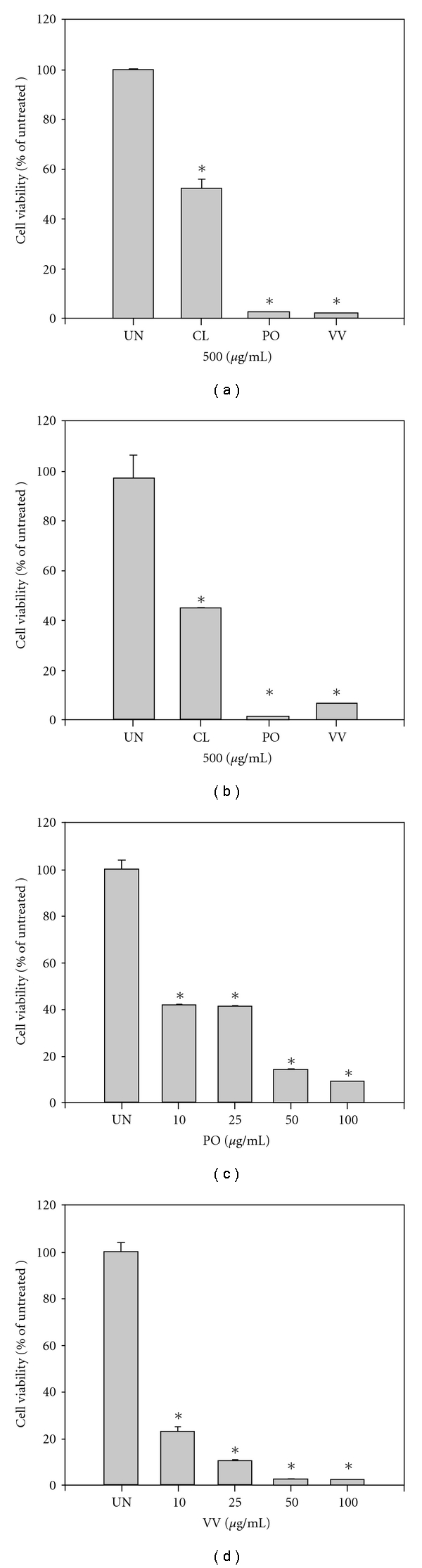
Cell viability analysis of CL, PO and VV protein extract-treated THP-1 and SW480 cells. (a) THP-1 and (b) SW480 cells were treated with CL, PO and VV protein extracts (500 *μ*g mL^−1^) for 24 h. SW480 cells were treated with 0, 10, 25, 50 and 100 *μ*g mL^−1^ of (c) PO and (d) VV protein extracts for 24 h. After treatment, cell viabilities were evaluated using the MTT assay. The values shown are the means ± SD (*n* = 5–8). *P < .05 (significant differences from the untreated group).

To evaluate the effects of protein extracts of CL, PO and VV on the cell viability of human monocytic leukemia THP-1 cells and human colorectal SW480 cells, a concentration of 500 *μ*g mL^−1^ was used for the MTT assay. As indicated in Figure 2(a), the viability of THP-1 cells was 52%, 3% and 3% after 24 h of treatment with CL, PO and VV, respectively. As indicated in Figure 2(b), the viability of SW480 cells was 48%, 2% and 7% after 24 h of treatment with CL, PO and VV, respectively. These results suggest that the degree of cell viability decrease observed for PO and VV was larger than that for CL in THP-1 and SW480 cells. We further selected PO and VV for evaluation of the effects of lower concentrations on the cell viability of SW480 cells. As indicated in Figure 2(c), the cell viability decreased to 39% after a 24-h treatment with 10 and 25 *μ*g mL^−1^ of PO protein extracts. The cell viability further decreased to 10 and 7% after treatment with 50 and 100 *μ*g mL^−1^ of PO protein extracts, respectively. Similarly to the decrease in cell viability induced by the PO protein extracts, the protein extracts of VV also decreased the viability of SW480 cells. As shown in Figure 2(d), treatment with 10 *μ*g mL^−1^ of VV protein extracts decreased cell viability by 20%; however, the decrease in cell viability was less than 10% after treatment with 25, 50 and 100 *μ*g mL^−1^ of VV protein extracts.

### 3.3. PO and VV Induced DNA Damage in SW480 Cancer Cells

**Figure 3:**
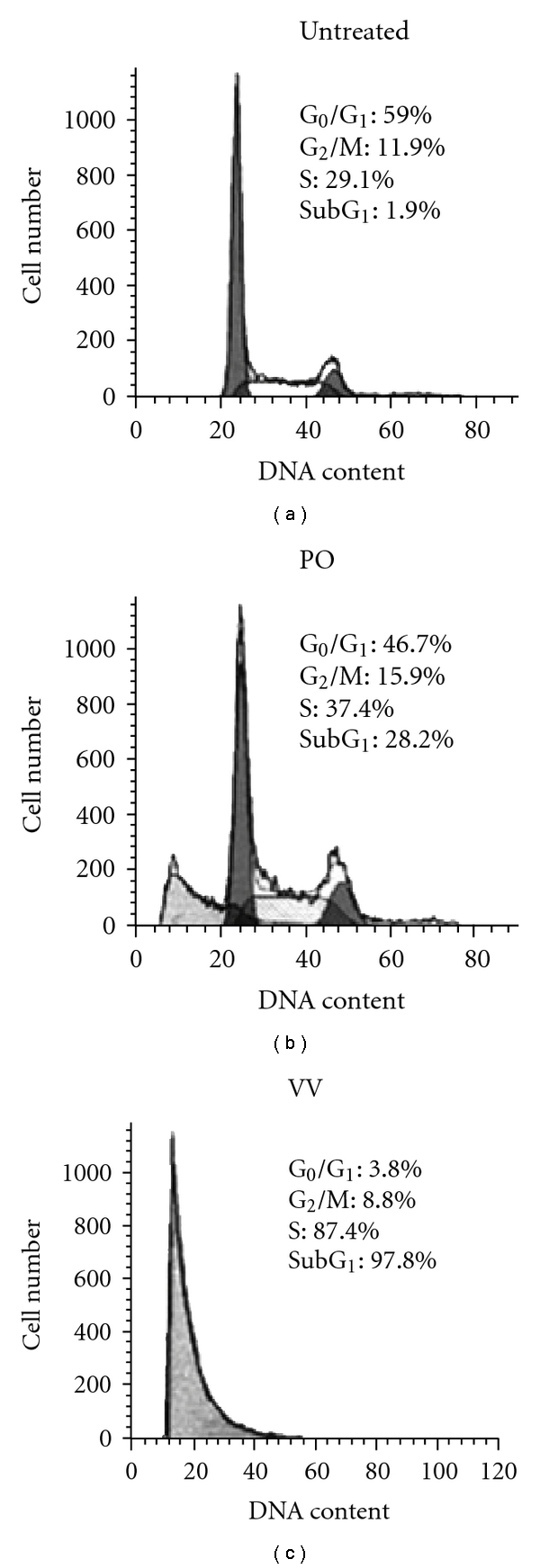
DNA damage analysis of PO and VV protein extract-treated SW480 cells. SW480 cells were treated with PO and VV protein extracts (100 *μ*g mL^−1^) for 24 h. After treatment, cells were fixed and then stained with propidium iodide, followed by flow cytometric analysis. Data in each panel show the percentages of cells in SubG_1_ (DNA-damaged cells), G_1_, S and G_2_/M phases of the cell cycle. These experiments were performed at least three times and a representative experiment is presented.

The DNA damage (SubG_1_) induced by CL protein extracts in SW480 cells was reported in our previous study [2]. The DNA damage induced by PO and VV protein-extract treatments in SW480 cells has not been extensively studied. We therefore evaluated whether the protein extracts of PO and VV could induce apoptosis. SW480 cells were exposed to 100 *μ*g mL^−1^ of PO and VV for 24 h, and the DNA damage of the cells was evaluated by flow cytometry. The total cells were separated into a damaged DNA group (cells in the SubG_1_ phase) and a normal cell cycle group (G_1_, S and G_2_/M phases). As shown in Figure 3, 59.0% untreated cells were in G_1_, 29.1% in S, 11.9% in G_2_/M and 1.9% in SubG_1_. The proportion of cells in SubG_1_ increased to 28.2% after treatment of SW480 cells with 100 *μ*g mL^−1^ PO protein extracts for 24 h. Following treatment with the protein extracts of VV (100 *μ*g mL^−1^) for 24 h, the proportion of cells in SubG_1_ markedly increased to 97.8% in SW480 cells.

### 3.4. PO and VV Induced ROS in SW480 Cells

**Figure 4:**
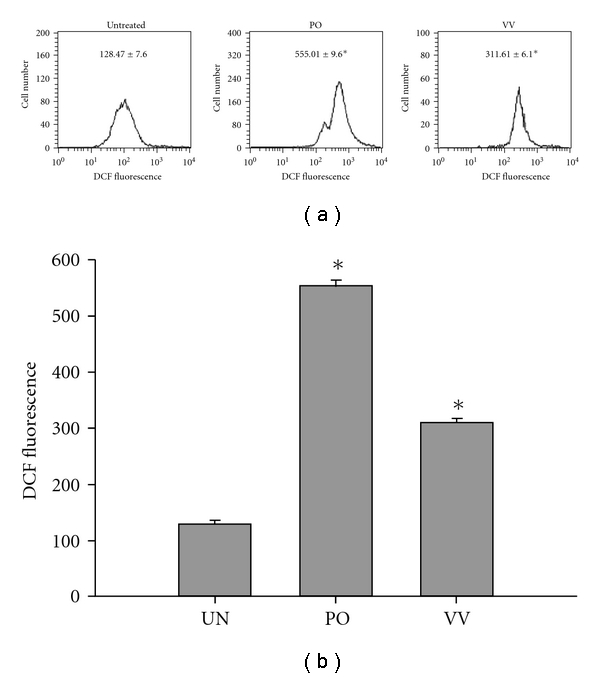
Intracellular ROS evaluation of PO and VV protein extract-treated SW480 cells. SW480 cells were exposed to PO and VV protein extracts (100 *μ*g mL^−1^) for 24 h. Production of intracellular ROS was detected by flow cytometry using DCFH-DA. The intracellular fluorescence of DCF was measured with a Becton-Dickinson FACScan flow cytometer. Data in each panel represent the DCF fluorescence intensity within the cells. The values shown are the means ± SD (*n* = 5–8 of individual experiments). *P < .05 (significant differences from the untreated group).

ROS are known to be important mediators of apoptosis and are induced by various stimuli, including many drugs. For this reason, intracellular ROS production was measured using a DCF fluorescence assay following treatment with PO and VV protein extracts. As indicated in Figure 4, SW480 cells were exposed to PO and VV protein extracts at 100 *μ*g mL^−1^ of for 24 h. The DCF fluorescence increased significantly from 128.47 ± 736 to 555.01 ± 9.6 and 311.61 ± 6.1 after treatment with PO and VV protein extracts, respectively.

### 3.5. PO and VV Induced GSH Depletion in SW480 Cells

**Figure 5:**
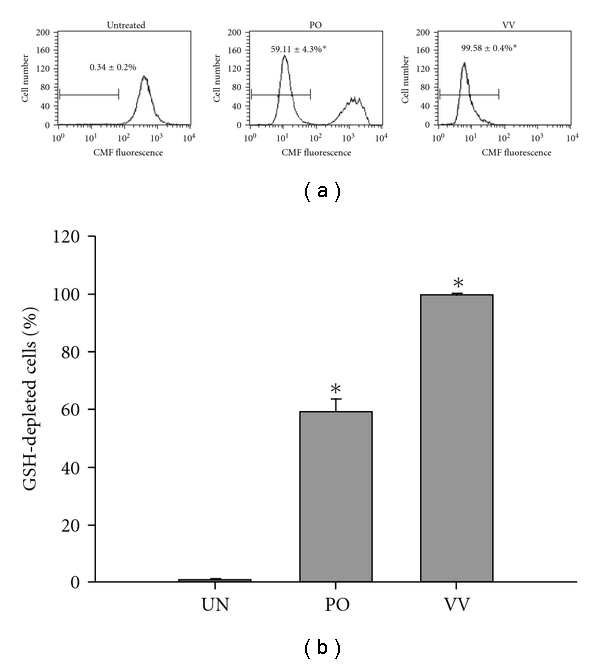
GSH depletion analysis of PO and VV protein extract-treated SW480 cells. SW480 cells were exposed to PO and VV protein extracts (100 *μ*g mL^−1^) for 24 h. After treatment, the cells were incubated with 25 *μ*M CMF-DA for 30 min in a 37°C CO_2_ incubator and fluorescence was then measured with a flow cytometer. Data in each panel represent the percentage of GSH-depleted cells with respect to the total cells. The values shown are means ± SD (*n* = 5–8). *P < .05 (significant differences from the untreated group).

GSH plays an important role in protection against oxidative stress-induced injury. Intracellular GSH depletion results in apoptosis. We therefore analyzed changes in the intracellular GSH level of SW480 cells after treatment with the protein extracts of PO and VV; we utilized flow cytometric analysis of CMF-DA fluorescence. SW480 cells were treated with protein extracts of PO and VV at 100 *μ*g mL^−1^ for 24 h. As shown in Figure 5, the fractions of cells with intracellular GSH depletion significantly increased to 59.11 ± 4.3 and 99.58 ± 0.4% after treatment with the protein extracts of PO and VV, respectively, as compared with untreated cells (0.34 ± 0.2%).

### 3.6. PO and VV Disrupted ΔΨ_m_ in the SW480 Cells

**Figure 6:**
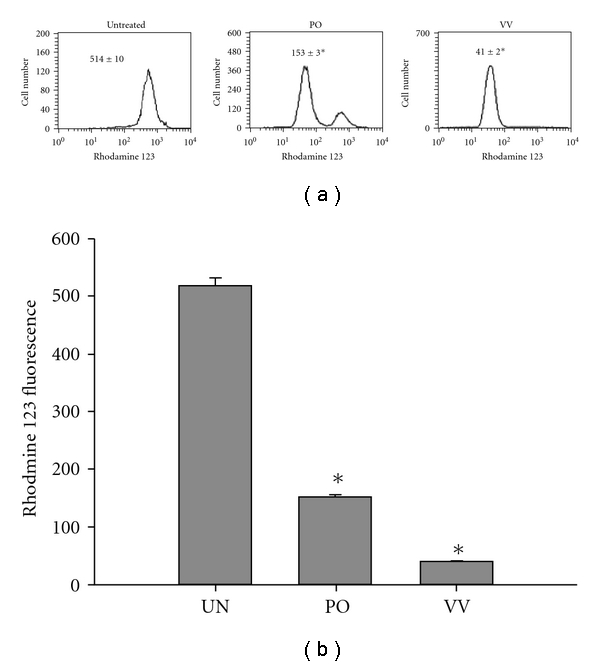
ΔΨ_m_ analysis of PO and VV protein extract-treated SW480 cells. SW480 cells were treated with PO and VV protein extracts (100 *μ*g mL^−1^) for 24 h. After treatment, the culture medium was replaced with a new medium containing 5 *μ*M rhodamine 123. Cultures were then incubated for 20 min in the dark. Values in each panel represent the rhodamine 123 fluorescence intensity within the cells. The values shown are means ± SD (*n* = 5–8). *P < .05 (significant differences from the untreated group).

Mitochondrial dysfunction usually triggers specific cellular signaling to induce apoptosis. Loss of ΔΨ_m_ is commonly used to measure mitochondrial dysfunction. To evaluate the effects of the protein extracts of PO and VV on ΔΨ_m_, SW480 cells were exposed to the protein extracts of PO and VV at 100 *μ*g mL^−1^ for 24 h. The ΔΨ_m_ was detected by staining of rhodamine 123, which specifically accumulated within the mitochondrial compartment in a ΔΨ_m_-dependent manner. As indicated in Figure 6, rhodamine 123 fluorescence was significantly decreased to 153 ± 3 and 41 ± 2 after treatment with the protein extracts of PO and VV, respectively, as compared with untreated cells (514 ± 10).

### 3.7. Evaluation of the Critical Pathway of Apoptosis Induced by PO and VV

**Figure 7:**
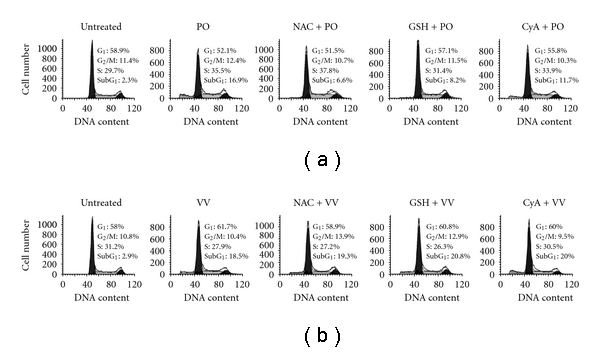
Evaluation of the critical mechanism in PO and VV protein extract-induced apoptosis. (a) The SW480 cells were treated with (a) PO and (b) VV protein extracts (0 (untreated) or 10 *μ*g mL^−1^) for 24 h, pretreated with 10 mM NAC, 10 mM GSH or 5 *μ*M CyA for 1 h, followed by (a) PO and (b) VV protein extracts (10 *μ*g mL^−1^) treatment for 24 h, and then analyzed for DNA damage. Data in each panel show the percentages of cells in the SubG_1_ (DNA-damaged cells), G_1_, S and G_2_/M phases of cell cycle. These experiments were performed at least three times and a representative experiment is presented.

To evaluate the critical pathway of apoptosis induced by the protein extracts of PO and VV, an extract concentration of 10 *μ*g mL^−1^ was used. As shown in Figure 7, 16.9% and 18.5% of cells were apoptotic (SubG_1_ phase) after treatment with the protein extracts of PO and VV for 24 h as compared with 2.3% and 2.9% of untreated cells, respectively. To evaluate the critical pathway, several agents were employed: (i) NAC, an intracellular GSH synthetic agent; (ii) GSH, were used to discover the GSH depletion pathway; and (iii) CyA, a mitochondrial permeability transition opening inhibitor, was used to determine the pathway involved in decreased ΔΨ_m_. SW480 cells were treated with these agents for 1 h before the addition of the protein extracts of PO and VV. As shown in Figure 7(a), NAC and GSH inhibited PO protein extract-induced apoptosis; the fractions of apoptotic cells decreased to 6.6 and 8.2%, respectively. CyA displayed a moderate inhibitory effect; the fraction of apoptotic cells was 11.7%.  Figure 7(b) shows that the apoptotic effect induced by the protein extracts of VV was not prevented by NAC, GSH or CyA; the fractions of apoptotic cells were 19.3, 20.8 and 20.0%, respectively.

## 4. Discussion

Currently, the prevalent choices for treating human cancer are surgical resection, general chemotherapy, gene therapy and radiation therapy. These treatments are costly and almost always performed in the hospital. It is necessary to develop new therapeutic strategies, such as integrative and complementary medicine, for cancer treatment. One approach, as highlighted in this study, looks for effective adjuvant substances from edible or medical mushrooms that are capable of activating the cellular apoptotic response in cancer cells [10]. Many edible and culinary mushrooms have become important medicinal and biotechnological species. For example, *Antrodia camphorata*, a unique mushroom of Taiwan, has been used as a traditional medicine for the prevention of various health-related conditions. It can be considered an efficient alternative phytotherapeutic agent or a synergizer for the treatment of cancer [11]. 4,7-dimethoxy-5-methyl-l,3-benzodioxole isolated from the fruiting body of *Antrodia camphorata* inhibits anchorage-independent proliferation and G_0_/G_1_ cell-cycle regulation in human colorectal carcinoma cells [12]. Another study reported that the basidiomycete mushroom *Agaricus blazei* Murrill, a mushroom native to Brazil, is traditionally used to treat many common diseases, such as atherosclerosis, hepatitis, hyperlipidemia, diabetes, dermatitis and cancer [13]. *Agaricus blazei* extract, which increases apoptosis via ROS-dependent JNK activation and inhibition of constitutive NF-*κ*B in leukemia THP-1 cells [14], has been reported to have anti-tumor and anti-mutagenic effects [15]. The water-extract of Chaga mushroom (*Inonotus obliquus*) has potent inhibitory effects on B16-F10 melanoma that include anti-proliferation, induction of differentiation and apoptosis [16]. It also induces G_0_/G_1_ arrest of the cell cycle and apoptosis in the human hepatoma Hep G2 cell line [17]. Several indian medicinal mushrooms, such as *Ganoderma lucidum*, *Phellinus rimosus*, *Pleurotus florida* and *Pleurotus pulmonarius*, have shown strong antioxidant and anti-cancer effects [18]. The results of the present study suggest that CL, PO and VV may be useful for integrative and complementary medicine by promoting apoptotic cell death in human colorectal cancer cells via induction of ROS production, GSH depletion and mitochondrial dysfunction.

In the present study, the protein extracts of CL, PO and VV were used to evaluate anti-cancer effects. The protein extracts of PO and VV induced marked apoptosis in SW480 cells (Figure 3). The apoptotic mechanism of the PO protein extract in SW480 cells was partially related to ROS production, GSH depletion and ΔΨ_m_ loss (Figure 7(a)). However, the apoptotic mechanism of the VV protein extract is not clear because NAC, GSH and CyA did not prevent VV protein extract-induced apoptosis (Figure 7(b)). This mechanism should be further studied in the future.

In 1963, related proteins and amino acids of PO were studied [19]. The first application of PO for antineoplastic activity utilized its polysaccharide [20]. Another study demonstrated that PO expresses marked antihepatoma and antisarcoma activities [21]. Treatment with the water extract of PO (150 *μ*g mL^−1^) induced phosphatidyl serine translocation from the inner membrane to the outer membrane after as little as 2 h and then induced apoptosis in human prostate cancer PC-3 cells [6]. Recently, the water-soluble polysaccharide extracted from the fruiting bodies of PO was shown to have obvious antitumor activity; they inhibited human HeLa cells in a concentration-dependent manner [11]. The water-soluble polysaccharide extracted from PO exhibited significantly lower cytotoxicity in human embryonic kidney 293T cells than in HeLa tumor cells when compared with 5-Fu [11]. These results suggest that the water-soluble polysaccharide extracted from PO may be a potential candidate for the development of a novel low-toxicity antitumor agent.

It is interesting that the protein extracts of VV had a stronger apoptotic effect than those of PO (Figure 3). Despite the lack of clear understanding of the detailed apoptotic mechanism of the protein extracts from VV, several other reports have observed anti-cancer effects for VV. The cold-alkali extracts and the polysaccharide of VV expressed antitumor activity [22–24]. Another study demonstrated that the lectin of VV exhibits immunomodulatory effects, stimulating the expression of Th1 cytokines and the proliferative activity of mouse splenocytes [25]. The lectin of VV also induces rapid expression of CD69, CD25, nuclear factor of activated T cells, IL-2 and proliferating cell nuclear antigen in a concentration- and time-dependent manner, leading to lymphocyte proliferation in a calcium-dependent manner [26]. The VV lectin also arrested S180 mouse sarcoma cell proliferation by blocking cell cycle progression in the G_2_/M phase via activation of the expression of the cyclin kinase inhibitors p21, p27, p53 and Rb [27].

**Figure 8:**
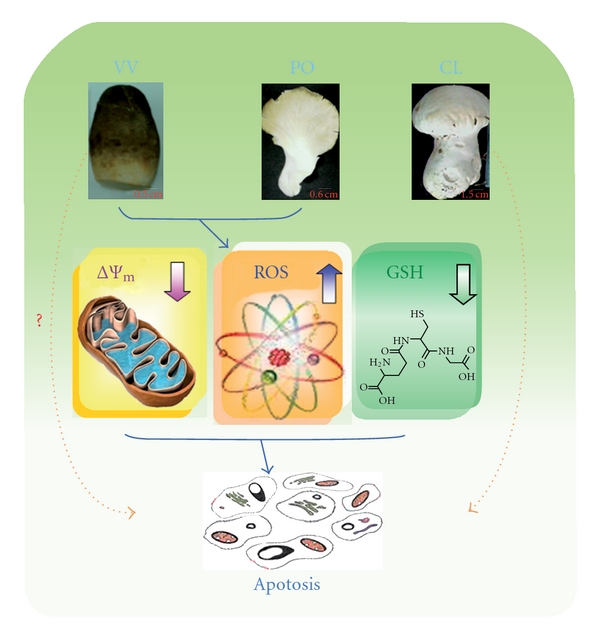
A hypothetical diagram for the anti-cancer effect of CL, PO and VV protein extracts.

The regulation of apoptosis is related to the susceptibility of cancer cells to chemotherapy. Many anticancer drugs can slow the progression of leukemia and other cancers through induction of apoptosis [28]. For this reason, apoptosis is considered to be a very important event for the development of anti-cancer drugs. The present results demonstrated that the protein extracts of PO and VV not only induced apoptosis but also resulted in ROS production, GSH depletion and ΔΨ_m_ loss. These phenomena are similar to effects observed for some clinical and pre-clinical anticancer drugs. For example, cisplatin can change the intracellular ROS and ΔΨ_m_ in human Hep G2 hepatoma cells [29]. Curcumin, a pre-clinical anticancer drug, can increase ROS, induce GSH depletion, decrease ΔΨ_m_, release cytochrome *c* and activate caspases to induce apoptosis in cancer cells [30]. Our previous study also demonstrated that the protein extracts of CL-induced apoptosis in human colorectal SW480 cancer cells through *γ*-glutamylcysteine synthase inhibition and GSH depletion [2]. ROS production, GSH depletion and ΔΨ_m_ loss are highly correlated with PO protein-extract-induced apoptosis in SW480 cells. Pretreatment with NAC, GSH and CyA did not affect the fraction of cells in the SubG_1_ phase after treatment with VV protein extracts; thus, the mechanism of VV-induced apoptosis is unknown. This is the first finding that the proteins of PO and VV extracted by ammonia sulfate show marked anti-cancer effects in human colorectal cancer cells and human monocytic leukemia cells. A hypothetical diagram that illustrates the anti-cancer effect of CL, PO and VV protein extracts is depicted in Figure 8.

## 5. Conclusions

The protein extracts of PO induced ROS production, GSH depletion and ΔΨ_m_ loss; these events were correlated with apoptosis in SW480 cells. The protein extracts of CL, PO and VV can be considered as a source of potential cancer therapeutic agents.
